# Draft genome report of *Enterobacter roggenkampii* strain MBBL_G6CM1 isolated from milk of a dairy cow with clinical mastitis

**DOI:** 10.1128/mra.00302-25

**Published:** 2025-06-12

**Authors:** Md. Sayedul Islam, Md. Morshedur Rahman, Naim Siddique, Zhokhar Dudayev, Mousumi Saha, M.Nazmul Hoque

**Affiliations:** 1 Department of Microbiology and Public Health, Gazipur Agricultural University, Gazipur, Bangladesh; 2 Molecular Biology and Bioinformatics Laboratory, Department of Gynecology, Obstetrics and Reproductive Health, Gazipur Agricultural University, Gazipur, Bangladesh; 3 Department of Surgery and Radiology, Gazipur Agricultural University, Gazipur, Bangladesh; Wellesley College, Wellesley, Massachusetts, USA

**Keywords:** *Enterobacter roggenkampii*, draft genome, dairy cow, clinical mastitis

## Abstract

We report the draft genome sequence of *Enterobacter roggenkampii* strain MBBL_G6CM1, isolated from the milk of a dairy cow with clinical mastitis. The assembled genome spans approximately 4.98 Mbp, comprising 130 contigs with 103.0× sequencing coverage and a guanine (G) and cytosine (C) content of 55.5%.

## ANNOUNCEMENT

Mastitis, an inflammatory disease of the mammary gland, poses a significant challenge in dairy production due to its detrimental impact on milk yield and quality (1). This condition results in substantial economic losses for dairy farmers, making it a critical concern for both animal health and farm profitability (2, 3). This disease is caused by a diverse group of well-known pathogens as well as by many opportunistic microbes (4, 5). Among these emerging opportunistic pathogens, *Enterobacter* species have notably risen in bovine clinical mastitis (CM) cases, including *Enterobacter cloacae* (12.6%) and *Enterobacter sakazakii* (1.1%) (6). *Enterobacter roggenkampii*, a gram-negative rod-shaped bacterium in the *E. cloacae* complex (7), has recently emerged as an opportunistic pathogen of clinical concern (8, 9). We present the draft genome sequence of *E. roggenkampii* strain MBBL_G6CM1, isolated from CM milk in a lactating cow, providing genomic insights into this emerging dairy-associated pathogen.

Milk samples were aseptically collected from a Holstein-Friesian cow diagnosed with CM, as confirmed by the California Mastitis Test (10), on a dairy farm in Dhaka (23.73°N, 90.38°E), Bangladesh. For bacterial enrichment, 100 µL of milk was inoculated into 10 mL of nutrient broth (in a sterile test tube) and incubated overnight at 37°C. The enriched culture was then streaked onto nutrient agar (HiMedia, India) and incubated at 37°C for 24 h. Subsequently, isolates were subcultured on MacConkey agar (HiMedia, India) and Chromogenic agar (Liofilchem Co., Italy), followed by overnight incubation at 37°C. Presumptive bacterial colonies were selected based on phenotypic characteristics: lactose-fermenting (pink) colonies on MacConkey agar and green colonies on Chromogenic agar (8, 11). Gram staining revealed gram-negative rods (7), and species-level identification was performed using the VITEK-2 system (v9.01) (12). Genomic DNA was extracted from nutrient broth cultures using the QIAamp DNA Mini Kit (QIAGEN, Germany), with purity/concentration checked using NanoDrop 2000 (Thermo Fisher, USA) (13). Libraries were prepared with the Nextera DNA Flex Kit (Illumina, USA) from 1 ng DNA and sequenced (2 × 250 bp) on an Illumina MiSeq platform. A total of 2,467,126 raw reads per file (forward/reverse) were quality filtered using Trimmomatic v0.39 (14) to remove Illumina adapters, known Illumina artifacts, and phiX reads, and quality checked using FastQC v0.12.1 (15). Reads containing Phred scores of 20 were assembled using SPAdes v3.15.0 (16). The draft genome was annotated using NCBI Prokaryotic Genome Annotation Pipeline v6.9 (17) and *Enterobacter*-specific CheckM v1.2.3 markers (18). Further genome annotations of the MBBL_G6CM1 included prediction of metabolic features, antimicrobial resistance genes (ARGs), virulence factor genes (VFGs), plasmids, and pathogenicity score through RAST server (19, 20, 20, 21, 21), PlasmidFinder v2.1.1 (22), and PathogenFinder v1.1 (21), respectively, analyzed with default parameters unless specified.

The draft genome of *E. roggenkampii* MBBL_G6CM1 (4.98 Mbp) from bovine CM reveals 26 VFGs, 21 ARGs, and 5 plasmid replicons, indicating significant pathogenic and antimicrobial resistance potential. The CM-associated *E. roggenkampii* isolates demonstrated significant pathogenic potential (PathogenFinder score: 0.715) and antimicrobial resistance dissemination capacity, highlighting its emerging threat to dairy herds and underscoring the importance of incorporating such pathogens into livestock surveillance programs. A summary of genome assembly and annotations is presented in Table 1.

**TABLE 1:** Key genomic features of *E. roggenkampii* strain MBBL_G6CM1

*E. roggenkampii*	Genomic features								
	Size (bp)	Coverage (×)	GC (%)	Contigs	*N*_50_ (bp)	CDSs	Protein coding genes	Genome accession	SRA accession no.
MBBL_G6CM1	4,978,347	103.0	55.5	130	158,410	4,806	4,718	JBLUTZ000000000	SRR32410675

## Data Availability

The draft genome sequence of *E. roggenkampii* strain MBBL_G6CM1 has been deposited in GenBank under accession JBLUTZ000000000 (BioProject PRJNA1225490), with raw sequencing data available in the SRA under accession SRR32410675.
